# Lentiviral Vector Purification Using Nanofiber Ion-Exchange Chromatography

**DOI:** 10.1016/j.omtm.2019.08.007

**Published:** 2019-08-30

**Authors:** Jelena Ruscic, Christopher Perry, Tarit Mukhopadhyay, Yasu Takeuchi, Daniel G. Bracewell

**Affiliations:** 1 Department of Biochemical Engineering, University College London, Bernard Katz Building, Gower Street, London WC1E 6BT, UK; 2 Division of Infection and Immunology, University College London, The Rayne Building, 5 University Street, London WC1E 6EJ, UK; 3 Advanced Therapies Division, National Institute for Biological Standards and Control, Blanche Lane, South Mimms, Potters Bar, Hertfordshire EN6 3QG, UK

## Abstract

Lentiviral vectors (LVs) are used in cell and gene therapies due to their ability to transduce both dividing and non-dividing cells while carrying a relatively large genetic payload and providing long-term gene expression via gene integration. Current cultivation methods produce titers of 10^5^–10^7^ transduction unit (TU)/mL; thus, it is necessary to concentrate LVs as well as remove process- and product-related impurities. In this work, we used a packaging cell line WinPac-RD-HV for LV production to simplify upstream processing. A direct capture method based on ion-exchange chromatography and cellulose nanofibers for LV concentration and purification was developed. This novel scalable stationary phase provides a high surface area that is accessible to LV and, therefore, has potential for high-capacity operation compared to traditional bead-based supports. We were able to concentrate LVs 100-fold while achieving a two-log removal of host cell protein and maintaining up to a 90% yield of functional vector.

## Graphical Abstract

**Figure:**
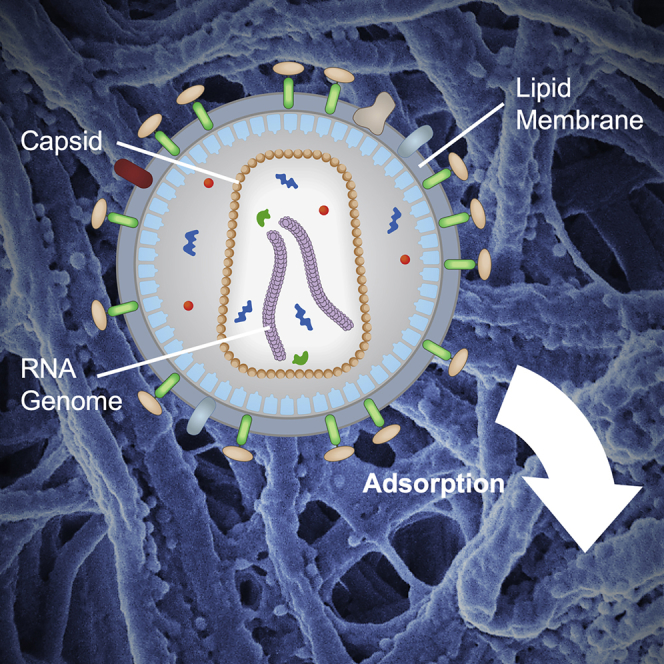

## Introduction

The encouraging results coming from ongoing gene therapy clinical trials and recently approved therapies, such as Kymriah,^1^ means there is a strong interest in processes for the scalable and cost-effective production and purification of viral vectors, which are considered to be a major roadblock to the commercialization of gene therapies.^2^ Current pricing for the commercially available gene therapies starts at half a million dollars per treatment, thus the number of patients with access to these therapies is small. In addition, there are significant potential applications of gene therapy in various chronic illnesses and in oncotherapy.

Lentiviral vectors (LVs), which, unlike other retroviral vectors, can transduce non-dividing cells, thus providing a wider range of potential applications, are important tools in cell and gene therapy. Currently, cell lines used for LV production provide titers of 10^5^–10^7^ transduction unit (TU)/mL,^3^ whereas 10^11^–10^12^ TU per patient^4^, ^5^ is being used for clinical applications. Therefore, it is necessary to extensively concentrate LV preparations as well as remove process- (e.g., serum proteins) and product-related impurities (e.g., non-infective vector), which can cause unwanted immune responses in patients.^6^ Small-scale purification and concentration can be achieved by ultracentrifugation, but there are several disadvantages to this approach: the method is time consuming, there are limited scale-up possibilities, some impurities can be co-purified that elicit an immune response, and the success of the process is strongly dependent on well-trained operator’s skills. Alternative methods that can provide scalable production include tangential flow filtration (TFF) and chromatography.

Currently, chromatography is dominated by porous bead stationary phases, which were designed for the purification of therapeutic proteins such as monoclonal antibodies (mAbs). This is not adequate for LV purification, since binding sites located within particle pores are typically not accessible to the considerably larger viral vectors; therefore, alternative stationary phases are necessary.^7^ Cellulose nanofibers are a new scalable purification platform. They are fabricated by electrospinning a non-woven fiber structure with diameters in the sub-micron range.^8^, ^9^, ^10^ The resulting adsorbent has an open structure with a large surface area accessible to viral vectors, and it allows operation at high flow rates due to mass transfer based on convection rather than diffusion, thus substantially shortening processing time. The application of nanofibers with different ligand densities in adenovirus type 5 vector purification has recently been reported.^11^

This work investigates whether nanofiber-based ion-exchange chromatography can provide a scalable LV purification process. Typically, a LV is produced via transient plasmid DNA (pDNA) transfection of adherent HEK293 cells in multiple T175 flasks or cell factories, where, 48–72 h post-transfection, lentivirus-containing mediums (LCMs) are harvested and processed. To circumvent problems associated with transient plasmid transfection and the consequent removal of the plasmid DNA as well as its sourcing problems, we used a continuous vector producer cell line, WinPac-RD-HV,^12^ and the Corning HYPERFlask system (total cell attachment surface area of 1,720 cm^2^, equivalent number of ten T175 flasks) to produce LVs used in our studies.

The WinPac-RD-HV cell line produces an LV with an RD-pro envelope protein derived from cat endogenous retrovirus RD114 and GFP reporter gene, thus avoiding problems associated with vesicular stomatitis virus G protein (VSV-G) cytotoxicity and allowing monitoring of LV infectivity via flow cytometry using the GFP reporter. Since LVs are structurally complex, in addition to the infectivity assay, we also measured LV recovery with several additional methods targeting different aspects of LV particle. The RNA genome was quantified via qRT-PCR using primers specific for the GFP gene,^12^ lentivirus-associated p24 capsid protein via ELISA, and RT enzyme via SYBR Green I-based product-enhanced RT (SG-PERT) assay.^13^, ^14^

## Results

### LV Production

**Figure 1:**
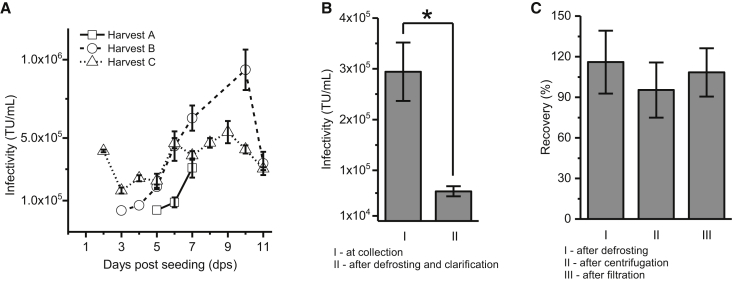
LV Production and Clarification (A) Optimization of LV production. In total 7.9 × 10^7^, 1.29 × 10^8^, and 2.37 × 10^8^ WinPac-RD cells were seeded in one HYPERFlask in harvest A, harvest B, and harvest C, respectively. SDs for harvests A and B represent duplicates and triplicates, while in harvest C all data points are all triplicates. (B) The effect of defrosting and clarification (low-speed centrifugation and filtration with Millex HP PES Express 0.45 μm) on infectivity of LV batch from harvest A (7 dps). At collection, n = 3; after defrosting and clarification, n = 12. (C) Infectivity recovery after defrosting and clarification (low-speed centrifugation and filtration with Millex HP PES Express 0.45 μm) in comparison to infectivity at harvest. Batches from harvest B were tested in defrosting experiments (n = 4) and batches from harvests B and C were tested in low-speed centrifugation (n = 14) and filtration with Millex HP PES Express 0.45 μm (n = 12) experiments. There are no statistically significant differences between samples obtained after defrosting and clarification compared to samples taken at harvest. Statistical analyses were performed using one-way ANOVA. *p < 0.05. Error bars represent mean ± 1 SD.

We performed three upstream process runs designated as harvest A, B, and C (Figure 1A) to study the impact of different seeding cell densities on LV titer. Because LV is released from the cells into the media, we were able to collect multiple batches of LCMs from a single HYPERFlask. In harvest A, 4.6 × 10^4^ cells/cm^2^ WinPac-RD cells were seeded under antibiotic selection, and 3 days post-seeding (dps) the first media exchange was performed using antibiotic-free complete media. LCMs from 4 dps showed no infectivity, while LCMs from 5 and 6 dps showed very similar low infectivity levels of 3.9 × 10^4^ TU/mL and 8.9 × 10^4^ ± 3.2 × 10^4^ TU/mL, respectively. The highest titer of 3.1 × 10^5^ ± 0.6 × 10^5^ TU/mL was obtained at 7 dps, and this LCM batch was stored in a 1-L bottle at −80°C.

In harvest B, we increased the amount of cells seeded in a single HYPERFlask to 7.5 × 10^4^ cells/cm^2^. Infective LV was detected earlier in harvest B (3 dps) than in harvest A (5 dps) (Figure 1A), and it continued to increase until 10 dps. LCM was not collected on 8 and 9 dps, thus the 10-dps batch contained LV produced over 72 h. There are insufficient data on LV half-life and stability at 37°C in the literature, especially LVs carrying other envelope proteins rather than VSV-G;^15^, ^16^, ^17^, ^18^ therefore, this is an element that needs to be addressed in the near future in order to develop efficient harvesting strategies. We observed a significant drop in infectivity at 11 dps in harvest B, which can be explained by cell detachment due to overgrowth observed while collecting the 10-dps batch, as well as containing LV produced in 24 h. Overall, higher total amounts of infective LV were obtained in harvest B (1.5 × 10^9^ TU) than harvest A (2.4 × 10^8^ TU), which can be explained by the higher density of seeded cells and larger volumes of collected LCMs.

In harvest C, we further increased the amount of seeded cells (1.4 × 10^5^ cells/cm^2^). Surprisingly, we obtained a relatively high infectivity immediately on 2 dps, which can be attributed to higher initial cell density and higher production over 48 h (Figure 1A). The infectivity dropped by 61% on 3 dps and stayed at approximately the same level during 4 and 5 dps. At 6 dps, the infectivity increased by 51%, and that level was maintained until 9 dps when it started to decrease. Overall, harvest C provided the highest amount of infective material (2.0 × 10^9^ TU) compared to two previous harvests. By comparing the three production runs, the importance of upstream process development (UPD) in order to obtain high titer and good quality LV material prior to embarking on downstream process development (DSP) is illustrated. Sanber et al.,^12^ who developed the Win-Pac-RDpro cell line, showed that high LV titers could be maintained for 4 days, with titers in the range of 10^6^ TU/mL when cells were seeded at a similar density to our harvest C. We obtained titers around 10^5^ TU/mL, which can be explained by the differences in infectivity assay execution where we did not perform the spinoculation step. Spinoculation has been shown to increase LV titers two to three times.^12^ Manceur et al.^19^ recently reported titers of 10^7^ TU/mL from a suspension-based inducible cell line obtained after medium composition optimization. This clearly shows that UPD is an area of extensive development, since it has a significant impact on LV infective titers as well as DSP due to optimized medium composition and quality of LV particles.

### LV Clarification

Harvest A 7-dps batch was defrosted overnight at 4°C. An infectivity assay performed after clarification (low-speed centrifugation and filtration with Millex HP PES Express 0.45 μm) revealed 80% loss of infectivity due to this freeze-thaw protocol (Figure 1B). To determine the origin of this loss, we studied the infectivity at each step. Bandeira et al.^20^ reported 83% losses in infectivity following a freeze-thaw cycle performed slowly on ice; therefore, our initial defrosting approach was a plausible culprit for this infectivity loss. In further production runs, we decided to store our LCMs in smaller volumes (100–200 mL) and defrost them quickly in water bath at 37°C. Batches from harvest B (5–7, 10 dps) were rapidly thawed in a water bath at 37°C, and their infectivity was compared to that prior to storage at −80°C (Figure 1C). The average recovery was 116% ± 23%; we conclude, therefore, that this approach is significantly better.

To investigate if low-speed centrifugation had an impact on LV infectivity, we tested all batches from harvest C and several batches from harvest B (5–7 dps, 10 dps). Infectivity recovery after this step was 95% ± 20% (Figure 1C); therefore, we can conclude that low-speed centrifugation has no negative impact on LV infectivity. Bandeira et al.^20^ have reported 70% recovery after low-speed centrifugation performed at 4°C under similar conditions (time and speed), while we performed ours at room temperature. Infectivity of several batches from harvest B (4–7 dps, 10 dps) and all batches from harvest C were also tested after filtration with Millex HP PES Express 0.45 μm to accommodate LV particle size of 100 nm. With no further optimization, our recovery compared to starting infectivity (before low-speed centrifugation) was 108% ± 17%, thus showing no loss of infectivity. This is comparable to 91% recovery obtained with scalable Sartopore 2 depth filters.^20^

### Tangential Flow Filtration

Tangential flow filtration (TFF) has previously been shown to concentrate infective LVs;^21^, ^22^ therefore, we initially explored this option. We tested four hollow fibers with different pore sizes (100, 300, 500, and 750 kDa molecular weight cut-off [MWCO]) in our preliminary diafiltration experiments with 20 mM Tris (pH 7.4). Hollow fibers with 300 and 500 kDa MWCO performed best in removing the majority of fetal calf serum (FCS) proteins and retaining LV (Figure S1). We opted to pursue the 500 kDa MWCO option.

**Figure 2:**
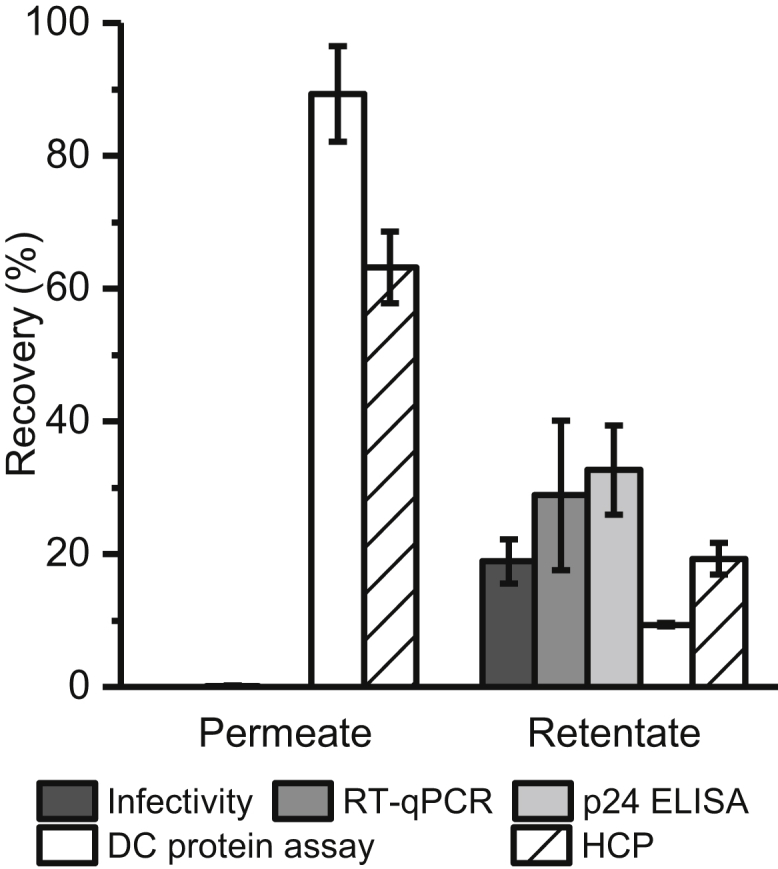
Recovery from TFF Concentration Experiments Batches from harvest B (5–7 dps) and mPES hollow fiber with 500 kDa MWCO were used (n = 5), where permeate and retentate samples were subjected to infectivity assay (n = 5), qRT-PCR (n = 5), p24 ELISA (n = 3), DC proteins assay (n = 2), and HEK293 HCP ELISA (n = 2). Error bars represent mean ± 1 SD.

We performed five concentration experiments with different LV batches, where we removed 89% ± 7% of the total protein (Figure 2, DC protein assay). When we used a more specific ELISA to track HEK293 host cell proteins (HCPs), we found 19% ± 2% was still in the retentate; but, since the LV membrane and core contain HCPs,^23^, ^24^ a certain amount is expected. Unfortunately, the infectivity assay showed that ∼80% of LV was lost, and similar results were obtained by both qRT-PCR and p24 ELISA. Although TFF has been previously successfully used to concentrate LVs,^21^, ^22^ the studies in question utilized LV pseudotyped with VSV-G envelope protein, transient transfection to produce LVs, and FCS in the diafiltration mix to preserve vector stability, while our source material was LV pseudotyped with RDpro envelope protein from a stable producing cell line. The influence of the source material (producer cell line and pseudotyped envelope) on LV stability while undergoing TFF concentration is an unexplored area. Nevertheless, one purification platform does not necessarily provide a satisfactory solution for all LV constructs,^25^ and alternative approaches need to be implemented; thus, we decided to pursue ion-exchange chromatography and cellulose nanofibers for LV concentration.

### Shear Impact on LV: Control Experiment

**Figure 3:**
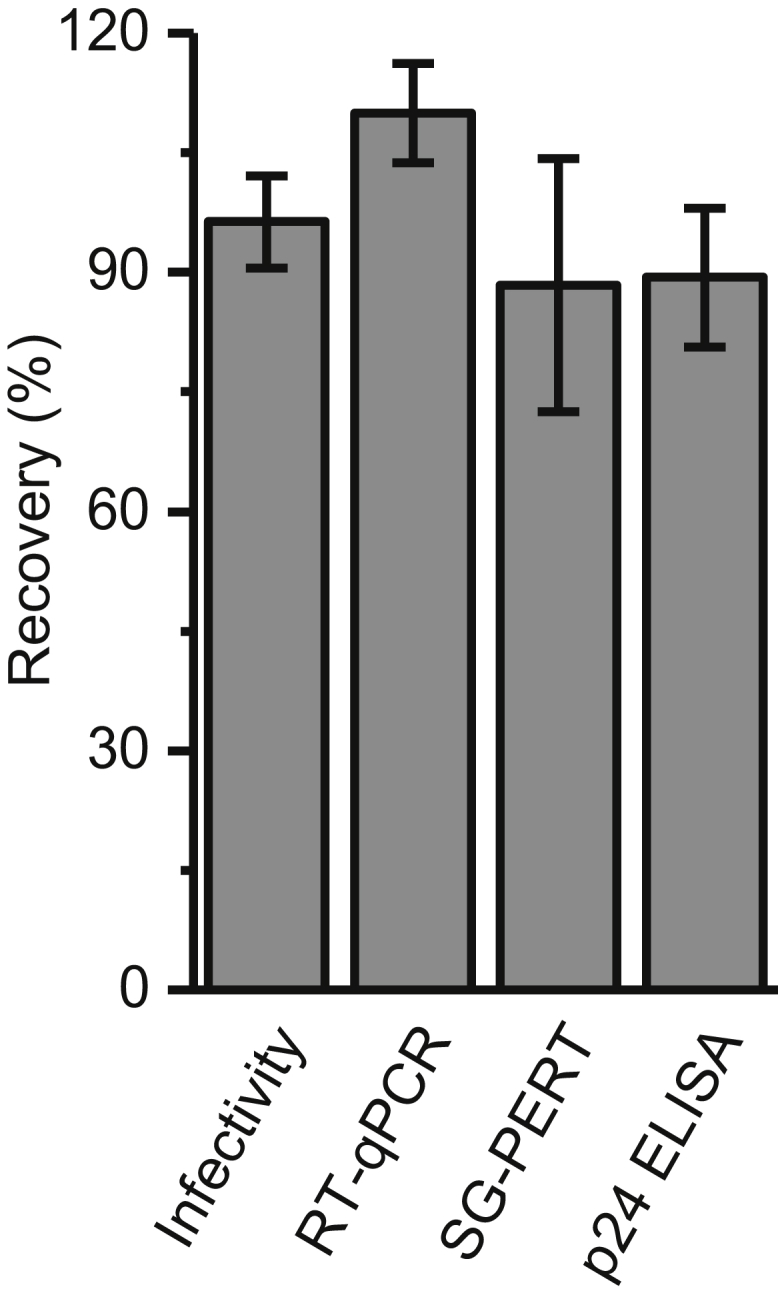
LV Recoveries Flow-through Experiments on Regenerated Cellulose Nanofibers Runs were done in triplicate and the 5-dps batch from harvest B was used. LV recovery was monitored by four assays: infectivity, qRT-PCR, SG-PERT, and p24 ELISA. Error bars represent mean ± 1 SD. Statistical analyses were performed using one-way ANOVA, and there were no statistically significant differences between results obtained with the four methods.

To eliminate the possibility of LV loss due to entrapment in the chromatography rig (AKTA Pure) or nanofibers themselves, as well as to take into account the impact of shear forces on LV stability, we performed an experiment in which we passed LCMs through the equipment and non-derivatized regenerated cellulose (RC) nanofiber adsorbent. Runs were done in triplicate and the whole system was washed with the loading buffer (flow rate 100 column volume [CV]/min, 10 mL/min). Due to the complexity of this viral vector, we employed multiple analytics to fully understand any potential losses thus LV recovery was monitored by four assays: infectivity, qRT-PCR, SG-PERT, and p24 ELISA (Figure 3). High recoveries were obtained with all four assays, with infectivity recovery of 96% ± 6% and qRT-PCR recovery of 110% ± 6%. SG-PERT and p24 ELISA gave very similar recoveries of 88% ± 16% and 89% ± 9%, respectively. In conclusion, all four assays show that there is no significant loss of LV in the fast protein liquid chromatography (FPLC) system and no entrapment of viable LVs in the nanofibers themselves, as well as no apparent impact of shear forces generated in the system on the functionality of the vector.

### Ion-Exchange Liquid Chromatography (IEX) Separation of LV

**Figure 4:**
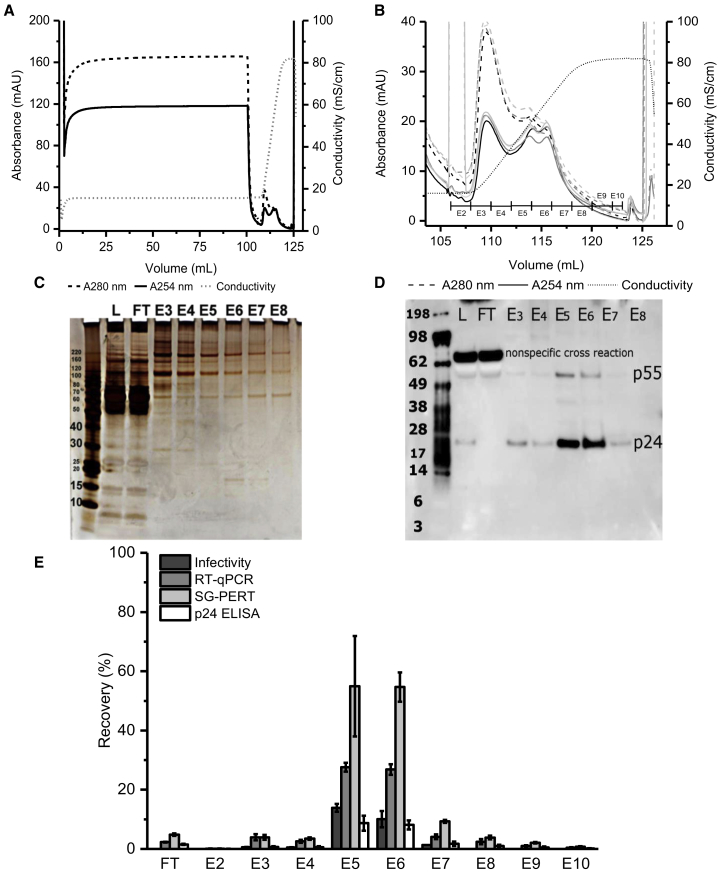
Initial LV Nanofiber IEX Experiments (A) Representative LV ion-exchange chromatographic profile performed using RCQ nanofiber. LCM from harvest B (6 dps),which was diluted four times with loading buffer and 100 mL was loaded onto the nanofiber. (B) A closer look at the elution profile from all four runs with corresponding (C) SDS-PAGE visualized with silver staining and (D) p24 western blot analysis of selected fractions from a single run using a polyclonal p24 primary antibody. (E) LV recovery analysis was performed for all four runs. L, load; FT, flow through; E, elution fraction. Error bars in (E) represent mean ± 1 SD.

LCM from harvest B (6 dps) was four times diluted with loading buffer and loaded onto the regenerated cellulose nanofibers derivatized with a RC quaternary amine (RCQ). Following a wash step, bound material was eluted using a linear gradient over 120 CV to a final concentration of 1 M NaCl. A representative chromatogram and elution profile are shown (Figures 4A and 4B). Silver-stained SDS-PAGE gel analysis of collected fractions (Figure 4C) revealed that the majority of protein content was found in the flow through, while smaller amounts were also present in early elution fractions (E3, E4), as seen by the A_254_/A_280_ ratio in the representative chromatogram (Figure 4B). Western blot analysis using a p24 polyclonal antibody (Figure 4D) showed that the majority of p24 was in elution fractions E5 and E6 (conductivity range of 38.12–63.67 mS/cm and NaCl concentration of 0.6–0.9 M). Although there were small traces of p24 in E3-E4 and E7-E8, when comparing the p24 band in the load to E5-E6, there was a clear concentration of p24 in the two elution fractions. In E5-E6, we also detected the p55 gag, a polyprotein that during LV maturation releases p24 and several other LV structural proteins. Surprisingly, we also found that this antibody recognizes a non-specific protein (>62 kDa) from FCS (Figure S2), which was present in the load but did not bind to the RCQ. We also re-examined our TFF diafiltration samples, and we found that the non-specific cross-reacting protein was present in both the permeate and retentate, thus demonstrating improved performance of RCQ compared to TFF in the reduction of this process-related impurity.

The infectivity assay based on GFP expression provides the most direct information on the quality of the purified LV material. To be infectious, LV particles need to have the following: (1) structurally preserved envelope and envelope protein in order to enter target cells; (2) functional genome and gene of interest (e.g., GFP); (3) RT enzyme and integrase in order to express the gene of interest; and (4) a functional nucleocapsid monitored via p24-lentivirus-associated p24 ELISA. Overall infectivity recovery was 27% ± 3% with 24% ± 4% in E5-E6 (Figure 4E). This recovery was similar to that obtained with TFF; therefore, additional quantitative analytics were used to provide further insight. qRT-PCR total recovery was 71% ± 5% with 2% ± 0.1% in the flow through and 54% ± 3% in E5-E6. Compared to TFF this is a significantly higher recovery.

SG-PERT quantifies RT encapsidated within the LV particle, and it has been shown to correlate with the infectivity.^13^, ^14^ The advantage of this assay is that it has a much broader dynamic range, higher sensitivity, and it can be completed in less than a day. SG-PERT total recovery compared to the load was 138% ± 20% with 5% ± 0.4% in the flow through and 110% ± 15% in combined fractions E5-E6. One of the reasons why total recovery was higher than 100% could be due to the presence of RT activity inhibitors in the load, thus underestimating its amount. Nevertheless, approximately 100% of recovery in E5-E6 is an encouraging result, which suggests that the losses seen in the infectivity assays are probably due to damage imposed on envelope protein or lack of functional genome and not to the inactivation of RT. Ideally we would have looked at the functionality of envelope protein and the integrity of LV membrane, but currently there are no commercially available assays that would give a quantitative answer to this question, highlighting the need for quick and reliable assays.

We used a lentivirus-associated p24 ELISA kit (Cell Biolabs) to quantify viral-associated p24 and, consequently, recovery of viral particles. The kit uses proprietary technology to separate virus-associated p24 from free p24, thus minimizing the overestimation of LV titer characteristic in standard p24 ELISAs. Total recovery compared to the load was 22% ± 6% with 2% ± 0.2% in the flow through and 17% ± 4% in E5-E6. Although the data are similar to the infectivity results and previously obtained TFF results, this assay is not able to distinguish between functional and non-functional viral particles.

### IEX Separation of LV with Direct Load

Since the salt concentrations of PBS used in our chromatography buffers and DMEM in LCM are similar, we directly loaded LCM onto the nanofibers. By taking this approach, we did not further dilute the FCS present in the load, which has been reported to have a beneficial impact on LV stability, although the exact mechanism of action is unclear.^26^ Initially we performed two chromatography runs where we loaded clarified LCM from harvest B (batch 3 dps, Figure S3). Total infectivity recovery was 62% ± 0.4% with 60% ± 3% in E5-E6 (elution within conductivity range 35.5–62.12 mS/cm, 0.6–0.9 M NaCl), which was double that of our previous results. The recovery from the other three assays, qRT-PCR, SG-PERT, and p24 ELISA, matched results obtained in the previous experiment. qRT-PCR total recovery was 72% ± 0.4% with 4% ± 0.1% in the flow through and 56% ± 2% in E5-E6. SG-PERT total recovery was 119% ± 16% with 12% ± 5% in the flow through and 92% ± 10% in E5-E6. P24 total recovery was 20% ± 1% with 1% ± 0.2% in the flow through and 16% ± 1% in E5-E6. The fact that infectivity doubled while other assays gave almost the same results from the previous experiment strongly implies that the way loading sample is prepared is relevant in preserving functionality of the envelope protein and/or the envelope itself.

**Figure 5:**
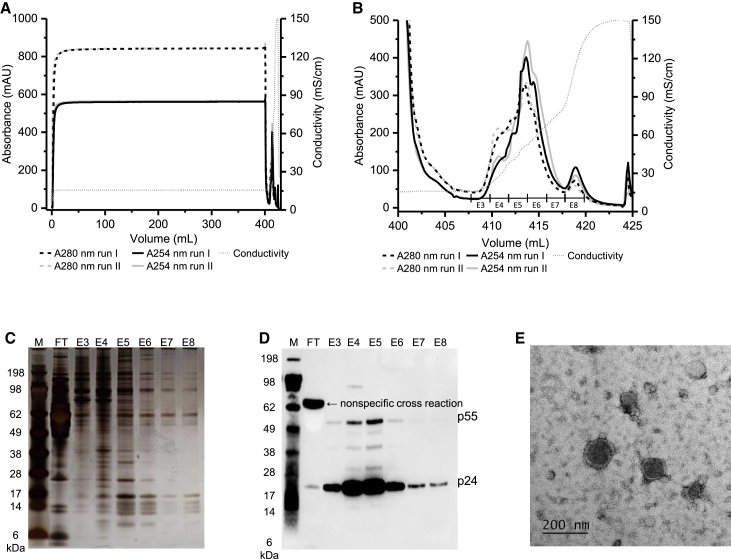
Final Scale-Up LV Purification Using Nanofiber IEX (A) Overlapping chromatograms of 400 mL LCM (harvest C, 7 and 9 dps) directly loaded onto a 0.1 mL RCQ nanofiber adsorbent module, (B) the elution profile, corresponding (C) SDS-PAGE visualized by silver staining, and (D) p24 western blot analysis of selected fractions from run II. (E) TEM image of elution fraction 5 (E5) from run II. L, load; FT, flow through; E, elution fraction.

Based on these initial encouraging results, we attempted to recover LVs from harvest C (7 and 9 dps). We performed two runs in which we loaded 400 mL of LCM directly onto the 0.1 mL RCQ nanofiber module (Figures 5A and 5B). Results from all four quantitative assays are shown in Table 1. Infectivity, qRT-PCR, and SG-PERT recoveries obtained in run I were very similar to our initial small-scale experiment with direct loading of LCM (Figure S3). Recoveries obtained in run II were significantly improved compared to run I and ranged from 94% to 120% for the three assays. The differences between run II and all other previous experiments was the fact that the infectivity assay was set up immediately after chromatography and SG-PERT was performed several hours later on the same day. In experiments with LV and monolith chromatography,^20^ initial infectivity recovery of 55% was reported. After optimization, which was immediate dilution of eluted material with Tris buffer infectivity, recovery was increased to >90%. We diluted our eluted LV in complete media rather than PBS or Tris due to the suggested stabilizing effect of FCS on LV.^26^ Based on this report, as well as our own results, immediate stabilization and proper storage of LVs after elution is crucial for maintaining high recoveries. The exact formulation is a matter of further research and is beyond the scope of this paper. Interestingly, high LV particle recoveries were obtained in both runs with p24 ELISA.

**Table 1:** Recoveries Obtained in Two Chromatographic Runs Performed by Loading 400 mL Harvest C Material to a 0.1-mL RCQ Nanofiber Adsorbent Module

	Recovery (%)							
	Infectivity		qRT-PCR		SG-PERT		p24 ELISA	
Run	I	II	I	II	I	II	I	II
FT	1.7	0.0	15.3	11.4	13.2	13.6	3.6	5.2
E3	0.7	0.7	4.4	3.6	1.7	11.3	0.7	1.7
E4	5.1	6.2	6.1	20.4	15.0	33.9	11.2	9.0
E5-6	41.0	85.4	40.8	62.7	61.8	58.7	56.1	95.0
E7	0.9	1.3	0.6	1.7	0.9	1.7	1.1	1.1
E8	0.3	0.0	0.2	1.4	0.6	1.4	0.3	0.6
Total	49.7	93.7	67.4	101.2	93.1	120.6	73.0	112.5

SDS-PAGE analysis of selected elution samples (Figure 5C) revealed the presence of several small protein bands with molecular weight between 10 and 20 kDa. Based on the observed pattern, it can be suggested that they are histone proteins,^27^ implying the presence of cellular DNA in the elution samples. Western blot analysis using a polyclonal p24 antibody (Figure 5D) revealed the presence of p24 in all elution fractions, with E4 and E5 having the highest amount. In addition, p55 as well as other intermediates in viral maturation (e.g., p41) was detected.^28^ Following overnight storage at 4°C, non-diluted aliquots of several elution fractions from run II were analyzed by transmission electron microscopy (TEM). A representative image of E5 (Figure 5E) revealed the presence of good-quality LV particles.

### HCP and DNA

**Figure 6:**
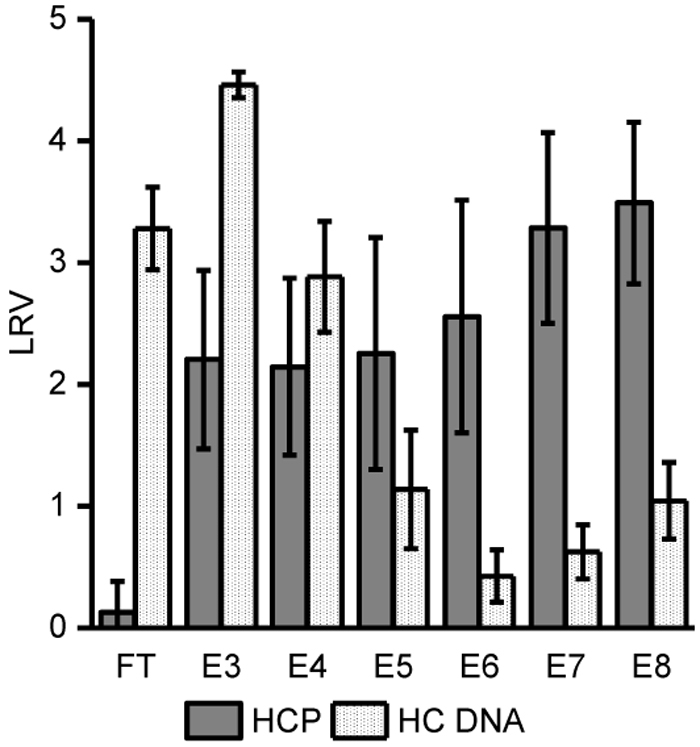
Removal of Host Cell Proteins and Host Cell DNA Determined by HEK293 HCP ELISA Kit (Cygnus Technologies) and Femto Human DNA Quantification Kit (Zymo Research), Respectively Fractions from three chromatographic runs were analyzed: one representative run from Figure 4, one from Figure S3, and run I (Figure 5). LRV, Log10 reduction value. Error bars represent mean ± 1 SD.

Fractions from three representative runs from three sets of experiments (Figures 4 and 5; Figure S3) were analyzed for HCPs and host cell DNA (HC DNA) content (Figure 6; Table S1). LCMs used in the three experiments originated from different LCM harvests and batches, which is reflected in HCP and HC DNA absolute content (two log reduction value [LRV] of HCP from elution fractions [E5-E6]). IEX chromatography step on RCQ nanofibers provides LV capture and 100-fold concentration directly from LCM and a commensurate removal of HCP. Due to the fact that HCPs are a part of LV membrane composition, an absolute removal of HCPs is not an expected outcome. Unfortunately, HC DNA is also being captured in LV elution fractions; therefore, further development of additional LV DSP steps, such as endonuclease treatment prior to the nanofiber IEX step, would be beneficial to the overall purification process.

## Discussion

In this work, we explored the application of a new type of stationary phase as a capture step for LV purification, where we achieved up to 90% recovery of biologically active/infective material. Unlike particle-based columns with intraparticle pore sizes designed for proteins, such a platform paves the way for scalable viral vector production to meet current and future market demands

Since our harvest titers were in the low range (low to mid 10^5^ TU/mL), we attempted to concentrate our LV material prior to chromatography by using TFF. This is a scalable technique that provides concentration as well as partial purification, and encouraging results were reported for LVs with VSV-G envelope proteins.^21^, ^22^ LVs with RDpro envelope proteins have been concentrated by TFF with recovery up to 30%.^12^ In our TFF concentration experiments, we did see a similar titer increase but 80% of infective material was lost, which was confirmed with additional analytics (qRT-PCR and p24 ELISA). Based on these three independent assays, we can assume that the loss of infectivity was probably due to LV particle entrapment within the hollow fiber pores; therefore, we decided to bypass this step and use nanofibers for the concentration of LV instead. Encouraging results were obtained with non-derivatized regenerated cellulose nanofiber, where we did not see any losses due to entrapment (Figure 3).

This may be a significant advantage compared to other non-traditional stationary phases, such as monolith and losses we experienced in TFF. Monoliths are cast as a single piece of material with interconnected channels and large enough diameters to accommodate viral particles, which provide mass transfer based on convection.^29^ Research on adenovirus type 5 (Ad5) vectors has shown that viral particles can be lost in the monoliths due to entrapment in the dead-end channels.^30^ We then performed experiments with nanofibers derivatized with quaternary (Q) amine ligands to create ion-exchange functionality. Although infectivity results were initially quite low, once we started applying undiluted LCM, infectivity doubled and reached ∼63%, which is similar to other LV chromatography recoveries reported.^20^, ^31^ In addition to that, nanofiber performed better in removing process-related impurities compared to TFF.

We were able to increase the recovered infectivity by immediately diluting the LV-containing fractions with media, which is similar to optimization strategies previously reported.^20^ This implies that LV stabilization is crucial in the first steps after capture by ion-exchange chromatography. Using this process, we were able to obtain LV particles with typical morphologies, as seen in TEM analysis of the elution fraction containing the highest amount of LVs (Figure 5E), and achieve two log removal of HCP (Figure 6). HCPs are an integral part of retroviruses and are detected in chromatography-purified viral vector particles;^32^ therefore, complete removal of HCPs is not possible. Unfortunately, unlike other reports,^20^, ^31^, ^33^ we did not achieve DNA removal from our LCM, thus further development would require an endonuclease step. The papers in question all used LVs with VSV-G envelope protein, and the impact of the different envelope proteins on LV chromatographic behavior has not been investigated. We know today that different viral serotypes and small changes in the virus structure can have a significant impact on the ion-exchange elution profile,^34^, ^35^ and it is highly likely that VSV-G and RDpro, as well as different HCP composition in the LV membrane, contribute to different strengths of interaction with ion-exchange ligands.

The absence of RT activity and lack of RNA genome and/or envelope proteins cause the formation of deficient/non-infectious vectors and contribute to heterogenicity of LV harvests in their production. Although minimizing the formation of these product-related impurities is part of the upstream optimization, ion-exchange chromatography has the potential to remove soluble retroviral envelope proteins and particles lacking the envelope protein from the biologically active retroviral particles with the envelope proteins.^36^ With the expansion of ligand types and chromatography modes, additional opportunities for the application of nanofibers in other viral vector DSP steps (e.g., polishing) are possible.

## Materials and Methods

### Cell Line and Culture Media

All cell lines were cultured at 37°C and 5% CO_2_. The HEK293T cell line was maintained in DMEM + GlutaMAX-I (Gibco), supplemented with 10% (v/v) fetal bovine serum (FBS) (Sigma) and 1× antibiotic-antimycotic (AntiAnti, Gibco). Lentivirus-producing cell line WinPac-RD-HR^12^ was maintained in DMEM + GlutaMAX-I supplemented with 10% (v/v), 1× AntiAnti, and antibiotics (1 μg/mL puromycin, 100 μg/mL hygromycin, 30 μg/mL phleomycin, and 10 μg/mL blasticidin). WinPac-RD-HR cells were expanded in T175 flasks under antibiotic selection and seeded in 10-layer HYPER*Flask* Cell Culture Vessels (Corning) to produce higher LV quantities necessary to carry out further experiments.

### LV Production, Storage, and Clarification

WinPac-RDpro cells were seeded in a single HYPER*Flask* using 560 mL of DMEM + GlutaMAX-I supplemented with 10% (v/v) FCS and 1× AntiAnti. In harvest A, 7.9 × 10^7^ WinPac-RDpro cells were seeded in a HPER*Flask* under additional antibiotic selection (1 μg/mL puromycin, 100 μg/mL hygromycin, 30 μg/mL phleomycin, and 10 μg/mL blasticidin). At 3 dps, antibiotic-containing media were discarded and fresh antibiotic-free complete media were added to the cells. Then 24 h later (4 dps), LCMs were collected and stored at −80°C in 500-mL bottles. This process was repeated every 24 h for the next 3 days. Aliquots from each day were stored at 4°C, and the infectivity assay was performed at the last day of collection (7 dps). Batch from harvest A collected on the 7 dps was defrosted at 4°C overnight followed by several hours at room temperature. This batch was then clarified by centrifugation (4,500 rpm, 15 min) and filtration (Millex HP PES Express 0.45 μm) at room temperature and used in diafiltration experiments.

Since detectable titers were obtained in harvest A batches without antibiotic selection, we removed antibiotics from further LV production runs in order to keep the cost down. In harvest B, 1.29 × 10^8^ WinPac-RDpro cells were seeded in one HYPER*Flask* with complete antibiotic-free media (DMEM + GlutaMAX-I supplemented with 10% [v/v] FCS). After 72-h incubation (3 dps), LCMs were collected and fresh antibiotic-free complete media were added to the cells. LCM was aliquoted in 150- or 250-mL bottles and stored at −80°C. This process was repeated every 24 h for the next 4 days. There was a break in collection on 8 and 9 dps, which was resumed at 10 and 11 dps. 1-mL aliquots from each batch were stored at 4°C, and the infectivity assay was performed several days later. LCM batches stored at −80°C were subjected to a quick defrost at 37°C in a water bath, followed by centrifugation (4,500 rpm, 15 min) and filtration (Millex HP PES Express 0.45 μm). Aliquots were taken after each step and analyzed by an infectivity assay. Batches from harvest B were used in TFF concentration experiments (5–7 dps), RC (5 dps), and initial RCQ experiments (6 dps).

In harvest C, 2.37 × 10^8^ WinPac-RDpro cells were seeded in one HYPER*Flask* with complete antibiotic-free media (DMEM + GlutaMAX-I supplemented with 10% [v/v] FCS). After a 48-h incubation period (2 dps), LCMs were collected and fresh media were added to the cells. LCM was immediately clarified by centrifugation (4,500 rpm, 15 min) and filtration (Millex HP PES Express 0.45 μm), after which 150- or 250-mL aliquots were stored at −80°C. At each step, 1-mL aliquots were taken and stored at −80°C. This process was repeated after 24 h for the next 9 days without any interruptions in collection. At the end of the collection, infectivity assays were performed with 1-mL aliquots, which were defrosted in heath block at 37°C. Batches from harvest C were used in the final RCQ experiments.

### TFF

TFF experiments were performed using a KrosFlo Research IIi System and mPES hollow fiber modules (Spectrum Labs). Four different MWCO sizes (100, 300, 500, and 750 kDa; D02-E100-05-N, D02-E300-05-N, D02-E500-05-N, and D02-E750-05-N, respectively, Spectrum Labs) were tested in triplicate in diafiltration (DF) experiments against 20 mM Tris (pH 7.4), with flow rate of 20 mL/min and manually controlled transmembrane pressure (TMP) of 1 psig. mPES hollow fiber with 500 kDa MWCO (D02-E500-05-N) was selected for further concentration experiments, where a flow rate of 30 mL/min and TMP of 1 psig were set.

### Chromatography

To determine whether AKTApure (GE Healthcare) itself (flow rate of 100 CV/min, 10 mL/min) and nanofiber membrane consisting of a non-derivatized RC (Puridify, now part of GE Healthcare) could have an effect on LV stability, flow-through experiments were performed. Runs were done in triplicate and membrane was washed with PBS (Gibco PBS tablets, cat. no. 18912014) + 0.0001% Tween 20 (pH 7.45). The RC column volume (CV) was 0.1 mL.

Harvest B 6-dps batch was 4 times diluted with loading buffer (PBS + 0.0001% Tween 20), and in total 100 mL was loaded to the 0.1-mL RCQ nanofiber membrane (0.1 mL CV, Puridify). Elution was done with a linear gradient over 120 CV with elution buffer (0%–100%) containing PBS (Gibco) and 1 M NaCl (Sigma). Runs were done in quadruplicate. The flow rate was 100 CV/min (10 mL/min). Undiluted 125 mL of harvest B 3-dps batch was loaded onto the RCQ nanofiber under the same conditions in duplicate.

In two independent runs (run I and run II), undiluted 400 mL from harvest C batches (7 and 9 dps) were loaded onto the RCQ nanofiber and eluted using a liner gradient (0%–50% elution buffer) over 100 CV, followed by a step gradient over 60 CV (100% elution buffer). Elution buffer contained PBS and 2 M NaCl. The flow rate was 200 CV/min (20 mL/min). Aliquots from elution samples from run I were diluted five times with complete media and stored at 4°C until infectivity assay and SG-PERT were performed 3 days later. Aliquots from elution samples from run II were also diluted five times with complete media, and infectivity assay was immediately started while an SG-PERT was performed several hours later. Aliquots of samples from both runs were stored at −20°C until the qRT-PCR and p24 ELISAs were performed.

### Infectivity Assay

Functional viral titer (TU/mL) of harvest batches, clarification and TFF samples, and chromatography fractions was determined by transduction of HEK293T cells on 12-well plate, followed by flow cytometric analysis of GFP expression by BD Accuri (BD Biosciences). Briefly, 3 × 10^5^ HEK293T cells were transduced with neat LVs or diluted LV samples (2-fold or 5-fold dilutions) in the presence of 8 μg/mL polybrene in a total of 500 μL. After 24 h, an additional 1 mL complete antibiotic-free medium was added to the cells, and after another 48 h (72 h in total) cells were analyzed for GFP expression after fixation in 4% paraformaldehyde (PFA) and 30-min incubation at 37°C to inactivate LVs. Titers were calculated from virus dilutions where 1%–20% of the cell population was GFP positive according to the following formula:

Titer (TU/mL) ={[No. of cells at transduction ×(% of GFP-positive cells/100)]/vector input volume}×dilution factor

### Vector Genome Recovery Determination Using One-Step qRT-PCR

A QIAamp Viral RNA Kit (QIAGEN) was used to isolate total RNA from the fractions. 2 ng luciferase control RNA (LUC) was added per sample immediately prior to isolation to account for discrepancies between samples during the isolation process and/or qRT-PCR. qRT-PCRs were performed with iTaq Universal SYBR Green One-Step Kit (Bio-Rad). 5 μL neat or diluted RNA was applied to the LV-GFP-specific^12^ qRT-PCR assays in duplicate, as well as to the LUC-specific^37^ qRT-PCR assay in 20 μL final volume. The primers’ final concentrations were 300 nM and cycling conditions were defined according to the manufacturer’s instructions. The qRT-PCR was performed on a CFX Connect Real-Time PCR Detection System (Bio-Rad). Average Ct values for LV-GFP were determined for each fraction and used to calculate LV genome concentrations via previously generated standard curves using StemMACS EGFP mRNA (Miltenyi Biotec). Recoveries were calculated as the percentage of virus genome present in the fractions in relation to the load. A buffer control and a non-template control (NTC) were included on each plate. Samples with Ct > 30 were considered to be negative. Normalization was performed according to the following formula:

$${\text{LV}\text{-}\text{GFP Ct}}_{\text{sample normalized}}=\;\left({\text{LUC Ct}}_{\text{buffer control}}{\text{– LUC Ct}}_{\text{sample}}\right){+\text{ LV}\text{-}\text{GFP Ct}}_{\text{sample}}$$

### LV RT Recovery Determination Using SG-PERT Assay

The SG-PERT assay was performed as described previously^13^, ^14^ with minor modifications. iTaq Universal SYBR Green One-Step Kit (Bio-Rad) components were used to perform the assay minus the iScript Reverse Transcriptase component of the kit. HIV Reverse Transcriptase (Merck) was used to build a standard quantification curve and MS2 RNA and MS2-specific primers were used.^14^ Prior to performing the assay, chromatography elution fractions were five times diluted in DMEM + GlutaMAX-I supplemented with 10% (v/v) FBS. Otherwise, neat samples were used.

### LV Particle Recovery Determination

Recovery of LV particles was determined with QuickTiter Lentivirus Titer Kit (Lentivirus-Associated HIV p24, Cell Biolabs), according to the manufacturer’s instructions.

### Protein and HC DNA Analysis

The protein composition of TFF samples and chromatographic fractions was analyzed by SDS-PAGE using NuPAGE 4%–12% Bis-Tris Protein Gels (Invitrogen), which were stained with SimplyBlue SafeStain Coomassie Brilliant Blue solution (Invitrogen). Selected gels were further stained with ProteoSilver Silver Stain Kit (Sigma-Aldrich). Western blots were performed using iBLOT 2 Dry Blotting System (Invitrogen) and iBlot 2 Transfer Stacks, polyvinylidene fluoride (PVDF) (Invitrogen). Membranes were blocked in SuperBlock T20 (PBS) Blocking Buffer (Invitrogen). Primary antibody was Rabbit polyclonal to HIV p24 (Abcam, ab63913, 1/2,500) and secondary antibody was Goat Anti-Rabbit immunoglobulin G (IgG) H&L (horseradish peroxidase [HRP]) (Abcam, ab205718, 1/20,000). Membranes were developed using SuperSignal West Pico Chemiluminescent Substrate (Thermo Scientific). Stained gels and membranes were documented using Amersham Imager 600 (GE Healthcare). DC protein assay (Bio-Rad) and HEK293 HCP ELISA kit (Cygnus) were used for protein quantification, according to the manufacturers’ instructions. HC DNA was quantified using Femto Human DNA Quantification Kit (Zymo Research), according to the manufacturer’s instructions.

### TEM

Selected elution fractions together with the load material used for separation were examined under TEM using the negative staining method. Samples stained with 1% aqueous uranyl acetate using the sequential drop method (2-min adsorption onto a plasma-cleaned carbon/formvar TEM grid, 2× 30-s washes in dH_2_O and 1 min in stain). Excess stain was removed from grid and samples were air-dried. Grids were images in a JEM2100 electron microscope (JEOL, UK) at 200 Kv under normal imaging conditions. Images were captured on a Gatan US4000 camera running digital micrograph 2 (GMS2) (Gatan, USA) at 10,000, 12,000, 25,000, and 50,000 magnifications with exposure times of 1–4 s.

### Statistics

Statistical analyses were performed using one-way ANOVA using OriginPro 2017 software. Tukey, Bonferroni, Dunn-Sidak, Fisher least significant difference (LSD), Scheffe’, Holm-Bonferroni, and Holm-Sidak tests were used, with significance level set at 0.05.

## Author Contributions

D.G.B. conceived the study. J.R. designed and executed experiments and drafted the manuscript. Y.T. provided cell lines used in the study and protocols for SG-PERT and the infectivity assay. C.P. assisted with assay and material transfer between labs and executed the final protein composition experiments. Research was performed in the laboratory of D.G.B. and T.M. with their oversight. J.R. and D.G.B. wrote the manuscript with contributions from all other authors.

## Conflicts of Interest

The authors declare no competing interests.

## Supplemental Information

Document S1. Figures S1–S3 and Table S1

Document S2. Article plus Supplemental Information
