# Supplementary material for: Lentiviral Vector Purification Using Nanofiber Ion-Exchange Chromatography
Source: Mol Ther Methods Clin Dev. 2019 Aug 30;15:52–62. doi: 10.1016/j.omtm.2019.08.007 (PMC6804883; doi:10.1016/j.omtm.2019.08.007)
Supplement: Document S1. Figures S1–S3 and Table S1 [file mmc1.pdf]

OMTM, Volume *15*

Supplemental Information

Lentiviral Vector Purification Using

Nanofiber Ion-Exchange Chromatography

Jelena Ruscic, Christopher Perry, Tarit Mukhopadhyay, Yasu Takeuchi, and Daniel G. Bracewell

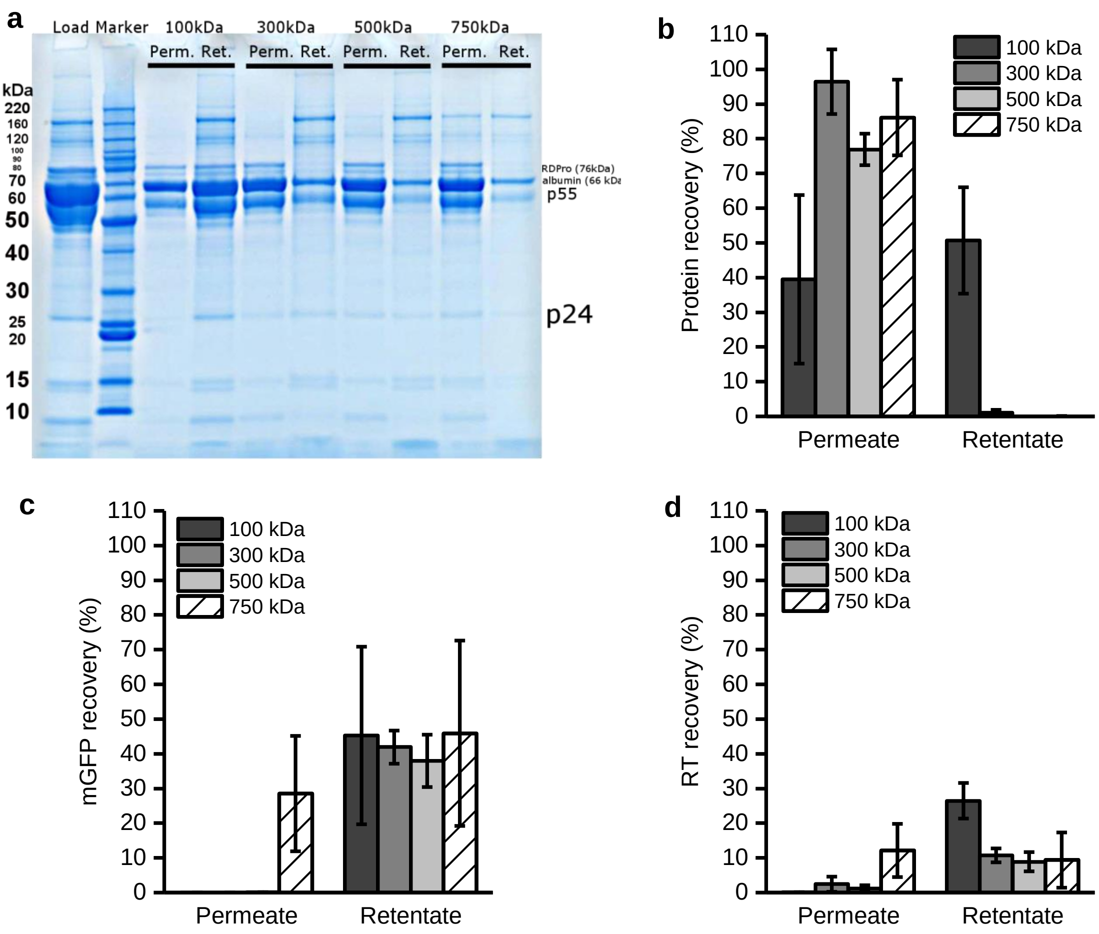

Fig. S1 | a, SDS-PAGE analysis, visualized by Coomassie stain, of diafiltration (DF) experiments using hollow fibre with different MWCO sizes (100kDa, 300kDa, 500kDa, and 750kDa). LV batch from harvest A (7 dps; Supplementary Fig 1a,b) was used in DF which was performed in triplicate. Permeate and retentate samples were tested with **b,** DC protein assay, **c**, RT-qPCR, and **d**, SG-PERT.

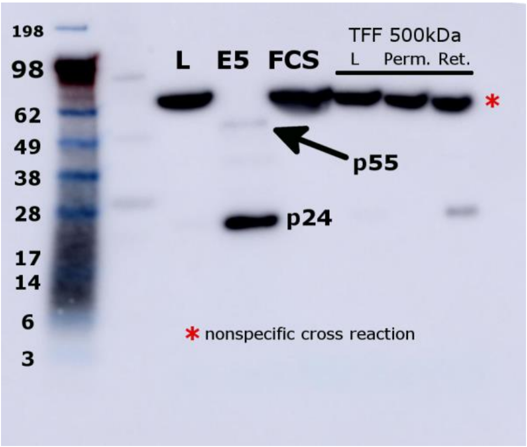

Fig. S2| p24 Western blot analysis of selected fractions performed using polyclonal p24 primary antibody: chromatography load (L), elution fraction (E5) and foetal calf serum (FCS). Samples from diafiltration experiments with 500kDa MWCO hollow fiber were also analysed (L – load, perm. – permeate, and ret. – retentate).

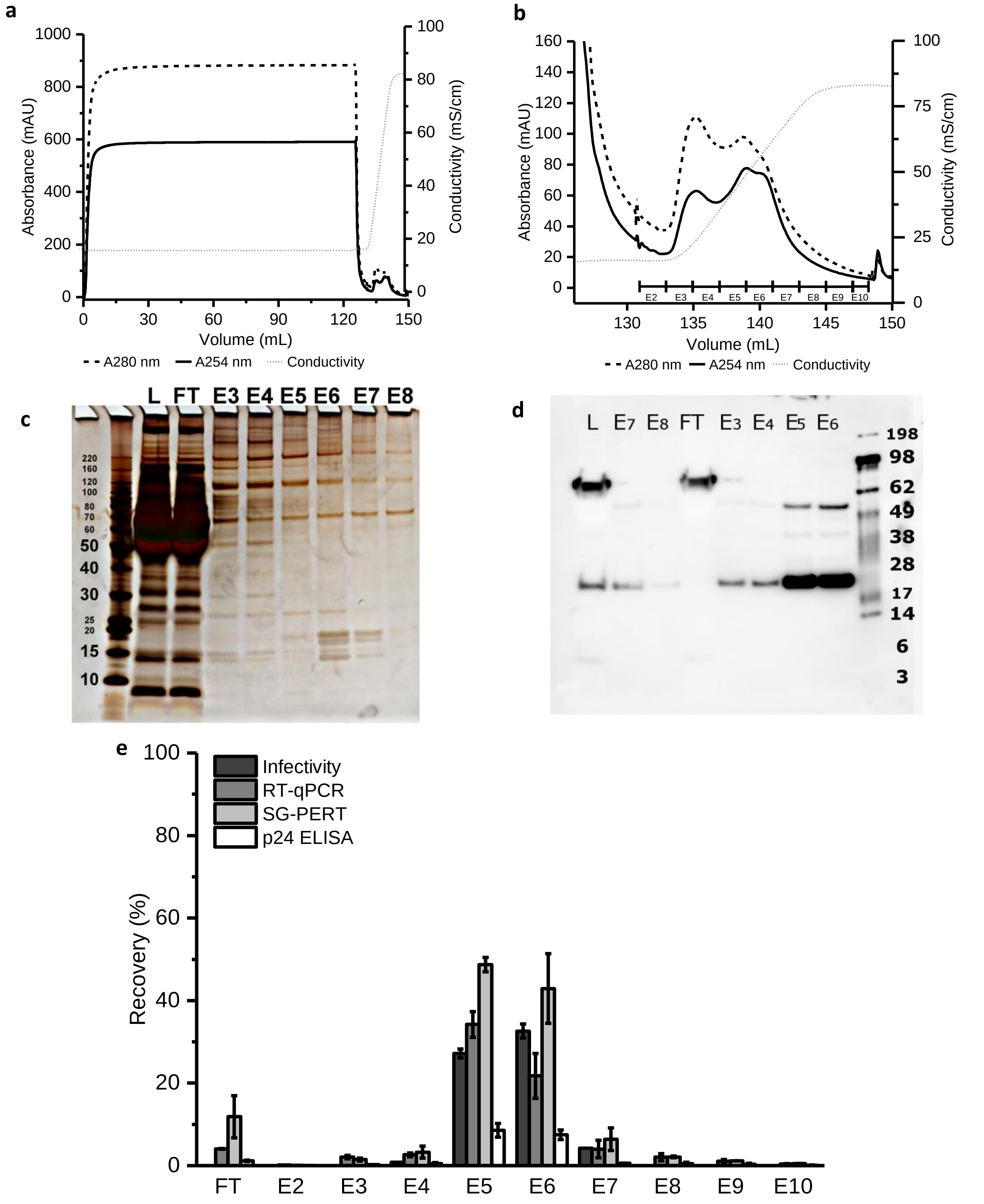

Fig. S3 | A) Representative chromatograms performed with 3 dps batch from harvest B directly loaded on to RCQ with B) closer look at the elution profile and corresponding C) SDS-PAGE visualised with silver staining and D) p24 Western blot analysis of selected fractions. Chromatography was done in duplicate and E) LV recovery analysis was performed for both runs. L – load, FT - flow through, E – elution fraction Error bars in e) represent mean ±1 SD

Table S1 Removal of host cell proteins (HCP) and host cell DNA determined by HEK 293 HCP ELISA kit (Cygnus Technologies) and Femto™ Human DNA Quantification Kit (Zymo Research) respectively. Fractions from three runs were analysed. Load from run I was four times diluted with the loading buffer (Supplementary Fig.4). Loads from runs II (Supplementary Fig. 6) and III (Figure 1) were not diluted

	HCP (ng/mL)			Total HCP (ng)			Log10 reduction value (LRV)
run	I	II	III	I	II	III	
load	73.6	57.5	781.1	$7.4x10^3$	$7.2x10^3$	$3.1x10^5$	
FT	45.2	25.1	$1.1x10^3$	$4.7x10^3$	$3.3x10^3$	$4.4x10^5$	0.1 +/- 0.3
E3	37.5	89.7	149.4	74.9	179.5	298.9	2.2 +/- 0.7
E4	44.2	100.5	171.6	88.3	201.0	343.3	2.1 +/- 0.7
E5	25.7	158.2	87.5	51.5	316.4	175.0	2.3 +/- 1.0
E6	11.6	84.0	45.1	23.2	167.9	90.1	2.6 +/- 1.0
E7	1.1	13.9	18.3	2.3	27.9	36.6	3.3+/- 0.8
E8	0.6	6.8	18.3	1.1	13.5	36.6	3.5 +/- 0.7

	HC DNA(ng/mL)			Total HC DNA (ng)			Log10 reduction value (LRV)
run	I	II	III	I	II	III	
load	104.8	275.3	540.0	$1x10^4$	$3.4x10^4$	$2.2x10^5$	
FT	0.1	0.1	0.1	12.8	15.3	57.0	3.3 +/- 0.3
E3	0.2	0.5	4.8	0.4	0.9	9.5	4.5 +/- 0.1
E4	3.3	13.8	467.8	6.7	27.6	938.5	2.9 +/- 0.5
E5	178.9	730.9	$2.8x10^4$	357.7	$3.4x10^4$	$5.7x10^4$	1.1 +/- 0.5
E6	$3x10^3$	$3.7x10^3$	$4.6x10^4$	$5.9x10^3$	$7.5x10^3$	$9.3x10^4$	0.4 +/- 0.2
E7	$2x10^3$	$4.5x10^3$	$1.5x10^4$	$4x10^3$	$9x10^3$	$2.9x10^4$	0.6+/- 0.2
E8	291.8	$1.1x10^3$	$2.2x10^4$	583.7	$2.2x10^3$	$4.5x10^4$	1.0 +/- 0.3
